# Dutch outcome in implantable cardioverter-defibrillator therapy (DO-IT): registry design and baseline characteristics of a prospective observational cohort study to predict appropriate indication for implantable cardioverter-defibrillator

**DOI:** 10.1007/s12471-017-1016-x

**Published:** 2017-08-07

**Authors:** M. van Barreveld, M. G. W. Dijkgraaf, M. Hulleman, L. V. A. Boersma, P. P. H. M. Delnoy, M. Meine, A. E. Tuinenburg, D. A. M. J. Theuns, P. H. van der Voort, G. P. Kimman, E. Buskens, J. P. G. Tijssen, N. Bruinsma, T. E. Verstraelen, A. H. Zwinderman, P. H. F. M. van Dessel, A. A. M. Wilde, on behalf of the DO-IT investigators

**Affiliations:** 1 Heart Centre, Department of Cardiology, Academic Medical Centre, Amsterdam, The Netherlands; 2 Department of Clinical Epidemiology, Biostatistics and Bio-informatics, Academic Medical Centre, Amsterdam, The Netherlands; 3 Clinical Research Unit, Academic Medical Centre, Amsterdam, The Netherlands; 4 Department of Cardiology, St. Antonius Hospital, Nieuwegein, The Netherlands; 5 Department of Cardiology, Isala Klinieken, Zwolle, The Netherlands; 6 Department of Cardiology, Division of Heart and Lungs, University Medical Centre, Utrecht, The Netherlands; 7 Department of Cardiology, Erasmus Medical Centre, Rotterdam, The Netherlands; 8 Department of Cardiology, Catharina Hospital, Eindhoven, The Netherlands; 9 Department of Cardiology, Medical Centre Alkmaar, Alkmaar, The Netherlands; 10 Department of Epidemiology, University Medical Centre Groningen, Groningen, The Netherlands; 11 Department of Cardiology, Thorax Centre Twente, Medisch Spectrum Twente, Enschede, The Netherlands

**Keywords:** Defibrillators, Implantable, Heart failure, Death, Sudden, Cardiac mortality, Risk assessment, Prospective studies

## Abstract

**Background:**

Implantable cardioverter-defibrillators (ICDs) are widely used for the prevention of sudden cardiac death. At present, both clinical benefit and cost-effectiveness of ICD therapy in primary prevention patients are topics of discussion, as only a minority of these patients will eventually receive appropriate ICD therapy.

**Methods/design:**

The DO-IT Registry is a nationwide prospective cohort with a target enrolment of 1,500 primary prevention ICD patients with reduced left ventricular function in a setting of structural heart disease. The primary outcome measures are death and appropriate ICD therapy for ventricular tachyarrhythmias. Secondary outcome measures are inappropriate ICD therapy, death of any cause, hospitalisation for ICD related complications and for cardiovascular reasons. As of December 2016, data on demographic, clinical, and ICD characteristics of 1,468 patients have been collected. Follow-up will continue up to 24 months after inclusion of the last patient. During follow-up, clinical and ICD data are collected based on the normal follow-up of these patients, assuming ICD interrogations take place every six months and clinical follow-up is once a year. At baseline, the mean age was 66 (standard deviation [SD] 10) years and 27% were women.

**Conclusion:**

The DO-IT Registry represents a real-world nationwide cohort of patients receiving ICDs for primary prevention of sudden cardiac death with reduced left ventricular function in a setting of structural heart disease. The registry investigates the efficacy of the current practice and aims to develop prediction rules to identify subgroups who will not (sufficiently) benefit from ICD implantation and to provide results regarding costs and budget impact of targeted supply of primary preventions ICDs.

**Electronic supplementary material:**

The online version of this article (doi: 10.1007/s12471-017-1016-x) contains supplementary material, which is available to authorized users.

## Background

Since its introduction in 1980, the implantable cardioverter-defibrillator (ICD) has become a generally accepted therapy for the prevention of sudden cardiac death (SCD) [1]. Its efficacy was demonstrated in both primary and secondary prevention settings [2–8]. Current international guidelines consider primary prevention ICD implantation as a class I indication in patients with New York Heart Association (NYHA) class II or III heart failure with ischaemic or non-ischaemic aetiology and left ventricular dysfunction (left ventricular ejection fraction (LVEF) of ≤35%). A class I indication is also recommended for patients with an LVEF ≤ 30% and NYHA I functional capacity [9, 10].

At present, both clinical benefit and cost-effectiveness of ICD therapy in patients with a primary prevention indication of SCD are being discussed. During mid-term follow-up, approximately two thirds of primary prevention ICD patients will never receive appropriate therapy for ventricular arrhythmias [3, 11–13]. They remain at risk of inappropriate shocks and device complications [14–16], which may impair recipients’ quality of life [17]. Furthermore, primary prevention randomised control trials (RCTs) were designed and started enrolling patients in the late nineties of the previous century. Both treatment of acute myocardial infarction (primary percutaneous intervention as opposed to thrombolysis) and treatment of heart failure have changed considerably. Today, the question arises whether primary prevention patients will be subject to the same number of appropriate shocks and will experience a similar relative risk reduction as patients in these ‘older’ RCTs.

Additionally, there is the ongoing debate whether LVEF values and NYHA functional classes are sufficiently accurate selection criteria for prognostic stratification of SCD risk. Consequently, there is a strong need to improve the current selection criteria for primary prevention ICD treatment in this setting [18–23]. In Europe alone, two prospectively designed cohort studies for improved risk stratification are ongoing [24, 25].

Cost-effectiveness studies show a wide variation in benefit, raising doubt about the efficiency, also regarding costs, of ICD therapy. ICD therapy appears to be only cost-effective in selected patient groups and seems to be related to the number of risk factors present for SCD [22, 26–31]. Risk stratification before ICD implantation is important from both a medical and an economic standpoint.

The DO-IT Registry aims to prospectively register the efficacy of implantation of primary prevention ICDs in order to develop prediction rules to identify subgroups who will not, or not yet, sufficiently or not at all, benefit from ICD implantation for primary prevention of SCD within two years of follow-up. What constitutes ‘sufficiently’ will be defined in a joint effort with a Dutch convenience sample of ethicists, patient representatives, cardiologists, health care insurers, health care policymakers, and key opinion leaders. Additionally, we aim to assess the costs and budget impact of targeted supply of ICD implantation in primary prevention patients. This article provides an overview of the DO-IT Registry design, a cohort description and describes the planned statistical analyses.

## Methods

### Design and study population

Since September 2014, the DO-IT Registry documents the current nationwide practice of ICD implantation in the primary prevention of SCD with reduced left ventricular function in a setting of structural heart disease. We planned to collect data on demographic, clinical, and ICD characteristics of 1,500 consecutively recruited patients in all 28 ICD implanting centres in the Netherlands. Currently, we are collecting follow-up data up to 24 months after inclusion of the last patient. The inclusion and exclusion criteria are shown in Tab. 1. The inclusion criteria are based on current guidelines (e. g. before 2014) for primary prevention ICD implantation [9, 10]. The LVEF assessment method was at the discretion of the local centre. The registry has been approved by the institutional review boards of all participating hospitals.

**Table 1:** Inclusion and Exclusion Criteria

*Inclusion Criteria*	
1	ICD implantation for primary prevention of sudden cardiac death
2	LVEF ≤35% and NYHA ≤ III or LVEF ≤30% and NYHA I
3	Life expectancy ≥1 year
4	≥40 days after myocardial infarction
5	≥90 days after revascularisation procedure
6	Optimal pharmacological heart failure treatment
*Exclusion criteria*	
1	Secondary prevention
2	ICD generator replacement
3	Inability or unwillingness to provide valid informed consent

*ICD* implantable cardioverter-defibrillator, *LVEF* left ventricular ejection fraction, *NYHA* New York Heart Association

### Data collection

Cardiologists, electrophysiologists, biostatisticians and health economists have jointly defined the relevant data set, in view of existing literature on possible predictors for SCD. Electronic case report forms (e-CRFs) were generated using an open source web-based software platform OpenClinica [32] in compliance with good clinical practice. By randomly generating a unique identification number, the anonymity of the patient is maintained. Data of the patient are collected from medical records and entered into the eCRF. All participating sites access the registry via a personal password protected login procedure and can only access the records of the patients of their own centre. We provided the participating centres with a sequential set of instructions for data entry and offered a training, if needed.

### Baseline and follow-up data

We collected baseline data on demographics, medical history and diagnostics, as well as implantation and device data. We collected clinical and ICD follow-up data, based on the normal follow-up of these patients assuming ICD interrogations take place every six months and clinical follow-up is once a year. Every six months, we register occurrence of ventricular arrhythmias and data regarding ICD therapies. ICD therapy is defined as anti-tachycardia pacing (ATP) and defibrillator shocks. During clinical follow-up, medical history, functional status, cardiovascular hospitalisations and performed procedures and diagnostics of the past year are entered into the registry. In addition, the participating hospitals report adverse events, defined as ICD related complications and deaths of any cause. Events such as death, arrhythmias, cardiovascular hospitalisations, cardiac interventions, diagnostic procedures and laboratory parameters between last follow-up visit and death are documented. Medical documentation around the time of death and ICD interrogations are collected, whenever possible. A more detailed list of the collected parameters is provided in the online supplementary material.

### Data and adverse event management

Verification rules and edit checks have been built into the system to validate completeness, accuracy and consistency of the dataset. Data quality is also maintained by online and on-site monitoring and continuous assessment of data quality during the export of datasets to statistical packages for subsequent analysis. A monitor plan is used which describes whether outcomes and variables need to be monitored entirely. This decision depends on the expected role of the variable in the prediction models, expected frequency of mistakes and the time needed to monitor the variable. The site investigator is required to maintain adequate records to ensure the manner in which the registry is conducted is well documented. The investigator should keep the records on file for a period of time specified by local law for the preservation of hospital documents. If requested, the investigator supplies the monitor with any required data.

A steering committee, represented by cardiologists of the participating hospitals, is responsible for the scientific integrity and the way that the study is conducted and advises the registry coordinating centre, when needed. A clinical event committee, consisting of independent cardiologists, assesses all ICD delivered therapies by analysing stored electrograms. The committee also determines whether deaths are of cardiac origin.

## Outcomes

The primary outcome measures of the DO-IT Registry are death and appropriate ICD therapy for ventricular tachycardia (VT) or ventricular fibrillation. Secondary outcome measures are inappropriate ICD therapy, death of any cause, hospitalisation for ICD related complications and cardiovascular reasons.

## Sample size/power

The registry has a target enrolment of 1,500 patients with a follow-up of at least two years. Based on the latest RCT’s and literature [3, 8] we expect to observe 150 (10%) patients with appropriate shocks. In combination with 150 fatalities (5% per year), these are sufficient to develop prediction rules.

## Data analysis

### Research aim 1: prediction rules

The incidence for the outcomes of appropriate ICD therapy and death are illustrated by Kaplan-Meier curves. Cox regression models with baseline characteristics of patients are developed to predict the outcomes of death or appropriate ICD therapy over time, considering the competing risk. The risk models will be developed according to the methods that have been used in the paper on the widely accepted ‘MADIT-II risk score’ for primary therapy with an ICD [33]. First, we will develop a model with important baseline characteristics, selected from previous publications on ICD mortality risk stratification and appropriate ICD therapy. We will consider age, gender, body mass index, LVEF, NYHA class, prior (<1-year) heart failure hospitalisation, history of atrial fibrillation and non-sustained VT, renal function, diabetes mellitus, smoking history, pulmonary disease and electrocardiographic parameters. Next, we will identify further factors, such as events during follow-up (complications and appropriate shock), that might improve prediction of the primary outcomes in exploratory analyses. The validity of the resulting predictor sets will be assessed by bootstrapping. A total of 200 bootstrap regression analyses will be performed for internal cross validation. The predictive values of the prediction models will be quantified using c‑statistics.

### Research aim 2: economic evaluation

A cost-minimisation analysis and a budget impact of targeted supply of ICD implantation will be performed. This economic evaluation will compare targeted supply of ICD implantation based on new prediction rules with current practice as reflected in the DO-IT Registry. Targeted supply, however, is not registered; no ICD implantation is withheld from patients who meet the current guidelines. That is why we are not able to observe what would have happened to these patients had they not received an ICD. To circumvent the missing data, the economic evaluation will be conducted as a modelling study, and assuming that not performing unnecessary ICD implantations does not affect health outcomes, only costs, except for temporal implantation related complications. These assumptions are realistic, but depend on the quality of the prediction rules. That is why we will develop prediction rules which can be applied in a way that will minimise the number of false negative predictions. Further details regarding the cost-minimisation and budget impact analysis are provided in the online supplementary material.

### Study population

**Fig. 1:**
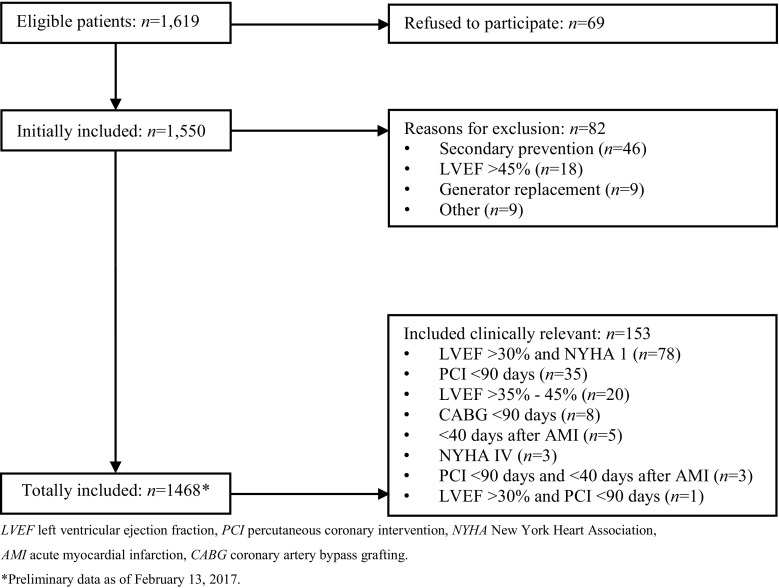
Flowchart of study participants

Between September 10th, 2014 and May 13th, 2016, 1,619 patients were assessed for eligibility. A flowchart of the study participants is presented in Fig. 1. Sixty-nine patients refused to participate. Data of 1,550 patients were initially entered into the registry. However, 82 patients were excluded because they did not meet the inclusion criteria for various reasons. Although 153 patients did not fully meet the inclusion criteria, we decided that these patients should remain in the registry because they received their ICD in context of primary prevention with a reduced LVEF in a setting of structural heart disease and were assumed to be a clinical relevant subgroup. Therefore, 1,468 patients in total were included in the DO-IT Registry.

### Baseline characteristics

The baseline characteristics are presented in Tab. 2. Overall, 1,468 patients received their first ICD (single-chamber or dual-chamber), subcutaneous ICD (S-ICD) or cardiac resynchronisation therapy defibrillator (CRT-D) for primary prevention of SCD (32%, 16%, 9% and 43%, respectively). Seventy-three percent of patients were men and the mean age at implantation was 66 (SD 10) years. The mean LVEF was 26% and more than half of the patients (61%) experienced NYHA class II heart failure symptoms. Most patients, 56%, had ischaemic heart disease. The remaining 641 (44%) patients were considered non-ischaemic.

**Table 2:** Comparison of patient characteristics of DO-IT Registry (date) with ICD arms of MADIT II and SCD-HeFT Trial

Patient characteristics	DO-IT (*n* = 1,461^a^)	DO-IT (*n* = 1,308, 153 patients excluded)	SCD-HeFT (*n* = 829)	MADIT II (*n* = 742)
Age (years)				
Mean (SD)	66 (10)	66 (10)		64 (10)
Median (IQR)	(68) (60–73)	(68) (60–73)	60 (52–68)	
Men (%)	73	72	77	84
LVEF				
Mean (SD)	26 (6)	26 (6)		23 (5)
Median (IQR)	27 (21–30)	26 (21–30)	24 (19–30)	
NYHA				
I (%)	14	9		35
II (%)	61	65	70	35
III (%)	22	23	30	25
(%)	<1			5
Unknown (%)	3	3		
Ischaemic cardiomyopathy (%)	56	54	52	100
Diabetes (%)	27	27	31	33
Hypertension (%)	43	43	55	53
Atrial fibrillation (%)	30	31	17	9

Data are presented as mean (standard deviation) or median (25th–75th percentiles)

*DO-IT* Dutch Outcome in ICD therapy,*ICD* implantable cardioverter-defibrillator, *SCD-HeFT* Sudden Cardiac Death in Heart Failure Trial, *MADIT II* Multicentre Automatic Defibrillator Implantation Trial-II, *IQR* interquartile range, *LVEF* left ventricular ejection fraction, *NYHA* New York Heart Association

^a^Preliminary data as of February 13, 2017.

## Discussion

In this article, we describe the design, cohort and planned analysis of the DO-IT Registry. This nationwide prospective registry aims to identify patients who will benefit most, or who will benefit the least, from ICD implantation. It further aims to assess the costs and budget impact of targeted supply of primary prevention ICDs. This is one of the largest prospective cohorts of primary prevention ICD patients, with a nationwide participation of all ICD implanting centres in the Netherlands. This research reflects current clinical practice and will provide real-world outcomes in the general population.

There are some differences between the baseline characteristics of the DO-IT patients and the patients included in the largest RCTs, MADIT II [8] and SCD-HEFT [3] (Tab. 2). Overall, DO-IT patients are older, more often female, and have higher LVEF. More patients have NYHA II symptomatic heart failure compared with MADIT II patients.

We enrolled 153 patients who did not fully meet the ACC/AHA/ESC Class I indication for primary prevention ICD implantation. The DO-IT Registry was designed to capture all primary prevention ICD patients. If the reason for ICD implantation was primary prevention for reduced LVEF in the setting of structural heart disease, patients were not excluded from the registry. Prior analyses of other primary prevention ICD registries also show that a proportion of patients do not meet trial inclusion criteria [34, 35]. Further analyses of these patients could clarify the event rate in this group, and might elucidate common risk factors that led to the decision to implant an ICD without a clear Class I indication.

## Study limitations

As expected, the registry has limitations [36, 37]. First, there is no control group with eligible patients who did not receive an ICD. However, the findings can be evaluated within the context of the previous RCTs or other registries. Second, there is a possibility of underreporting or misreporting, because data in registries are often collected more passively and monitored less strictly compared with RCTs. Third, patients should be included consecutively. However, the inclusion of patients was not controlled. For instance, sites may have been unable to enrol all eligible patients or simply have forgotten to register one. In addition, physicians may have been more likely to exclude patients with uncertain benefit of ICD implantation, which results in selection bias. However, we built in quality and control checks and online and on-site monitoring to ensure that the data is representative and of adequate quality.

Some recent articles are not discussed in the content of this paper because they were published later than the setup of the DO-IT Registry [38–40]. However, these studies also emphasise the need for improved selection of patients for primary ICD therapy since most patients do not experience benefit.

Although the DO-IT Registry is designed and funded for an initial follow-up duration of two years after inclusion of the last patient, other studies show that patients might benefit from primary prevention ICD therapy with follow-up longer than two years [11, 41]. However, additional post cohort projections and hypothetical modelling are anticipated. Moreover, the DO-IT Registry intends to extend the observation period if cooperation of the participating hospitals and additional funding can be arranged.

## Conclusion

At present, the indication for primary prevention defibrillators may not be optimal. Many patients do not receive appropriate ICD therapy, but are at risk of inappropriate shocks, device complications, or die without ever needing their ICD. The DO-IT Registry is a large nationwide cohort, consisting of patients receiving ICDs for primary prevention of SCD. Despite the limitations, we expect that the DO-IT Registry will identify subgroups of patients who will not, or not yet, sufficiently benefit from ICD implantation for primary prevention. We also expect that the registry will provide results regarding the costs and budget impact of targeted supply of primary preventions ICDs. Because of real-life data, the findings of this study should be representative and are likely to affect the current risk stratification of SCD.

## Caption Electronic Supplementary Material

The author’s affiliations and complete list of investigators is provided in the online supplementary material. In addition, more detailed infomation is given regarding the collected parameters and planned data analysis for the economic evaluation
