# Supplementary material for: Dutch outcome in implantable cardioverter-defibrillator therapy (DO-IT): registry design and baseline characteristics of a prospective observational cohort study to predict appropriate indication for implantable cardioverter-defibrillator
Source: Neth Heart J. 2017 Aug 7;25(10):574–80. doi: 10.1007/s12471-017-1016-x (PMC5612865; doi:10.1007/s12471-017-1016-x)
Supplement: Supplementary file 1 — The author’s affiliations and complete list of investigators is provided in the online supplementary material. In addition, more detailed infomation is given regarding the collected parameters and planned data analysis for the economic evaluation [file 12471_2017_1016_MOESM1_ESM.docx]

**Supplementary material DO-IT registry**

This supplementary material has been provided by the authors to give readers additional information about their work

Supplement to: van Barreveld M, Dijkgraaf MGW, Hulleman M et al. Dutch outcome in implantable cardioverter defibrillator therapy (DO-IT): registry design and baseline characteristics of a prospective observational cohort study for prediction of appropriate indication for implantable cardioverter defibrillator. Neth Heart J

Last update April 20, 2017

**Table of contents**

[List of registry coordination centre members 3](#_Toc470249539)

[List of Steering Committee members 4](#_Toc470249540)

[List of Clinical Event Committee members 5](#_Toc470249541)

[List of investigators 6](#_Toc470249542)

[List of collected parameters during the registry 7](#_Toc470249543)

[Economic Evaluation Analysis 8](#_Toc470249544)

[References 9](#_Toc470249545)

**List of Registry Coordination centre members**

A.M.M. Wilde^1^ (principal investigator), M. van Barreveld^1,2^, N. Bruinsma^1^, P.D.H.M. van Dessel^3^, M.G.W. Dijkgraaf^4^, M. Hulleman^1^, T.E. Verstraelen, A.H. Zwinderman^2^.

Affiliations:

^1^Heart centre, Department of Cardiology, Academic Medical Centre, Amsterdam, The Netherlands; ^2^Department of Clinical Epidemiology, Biostatistics and Bio-informatics, Academic Medical Centre, ^3^Department of Cardiology, Thorax Centre Twente, Medisch Spectrum Twente, Enschede, the Netherlands; ^4^Netherlands; ^4^Clinical Research Unit, Academic Medical Centre, Amsterdam, The Netherlands.

# **List of Steering Committee members**

A.A.M. Wilde^1^ (chair), L.V.A. Boersma^2^, E. Buskens^3^, P.P.H.M. Delnoy^4^, P.H.F.M. van Dessel^5^, G.P. Kimman^6^, M. Meine^7^, D.A.M.J. Theuns^8^, A.E. Tuinenburg^7^, P.H. van der Voort^9^.

Affiliations: ^1^Heart centre, Department of Cardiology, Academic Medical Centre, Amsterdam, The Netherlands; ^2^Department of Cardiology, St Antonius Hospital. Nieuwegein, the Netherlands; ^3^Department of Epidemiology, University Medical Centre Groningen, Groningen, the Netherlands; ^4^Department of Cardiology, Isala Klinieken, Zwolle, the Netherlands; ^5^Department of Cardiology, Thorax Centre Twente, Medisch Spectrum Twente, Enschede, the Netherlands; ^6^Department of Cardiology, Medical Centre Alkmaar, Alkmaar, the Netherlands; ^7^Department of Cardiology, Division of Heart and Lungs, University Medical Centre, Utrecht, the Netherlands; ^8^Department of Cardiology, Erasmus Medical Centre, Rotterdam, the Netherlands; ^9^Department of Cardiology, Catharina Hospital, Eindhoven, the Netherlands.

# **List of Clinical Event Committee members**

F.A.L.E. Bracke^1,^ R.E. Knops^2^, A.H. Maass^3^, M.F. Scholten^4^.

Affiliations: ^1^Department of Cardiology, Catharina Hospital, Eindhoven, the Netherlands; ^2^Heart centre, Department of Cardiology, Academic Medical Centre, Amsterdam, The Netherlands; ^3^Department of Cardiology, Thorax Centre, University Medical Centre Groningen, Groningen, the Netherlands; ^4^Department of Cardiology, Thorax Centre Twente, Medisch Spectrum Twente, Enschede, the Netherland.

# **List of investigators**

Listed in alphabetical order by institution.

Academic Medical Centre (A.A.M Wilde); Albert Schweitzer Hospital (M.W.F. van Gent); Amphia Hospital (S. Strikwerda); Canisius-Wilhelmina Hospital (L.H.R. Bouwels); Catharina Hospital (P.H. van der Voort); Erasmus Medical Centre (D.A.M.J. Theuns); Flevoziekenhuis (N.R. Bijsterveld); Isala Klinieken (P.P.H.M. Delnoy); Haga Hospital (R. Abels); Kennemer Gasthuis (R. Tukkie); Leiden University Medical Centre (L. van Erven); Maasstad Hospital (M. Firouzi); Maastricht University Medical Centre (K. Vernooy); Martini Hospital (L.H. Takens); Haaglanden Medical Centre (R.W. Grauss); Medical Centre Alkmaar (G.P. Kimman); Medical Centre Leeuwarden (A.E. Borger van der Burg); Medisch Spectrum Twente (M.F. Scholten); Onze Lieve Vrouwe Gasthuis (M. Khan); Rijnstate Hospital (R. Derksen); Scheper Hospital (M.W. Vet); St. Antonius Hospital (L.V.A. Boersma); Tweesteden Hospital (J.W.M.G. Widdershoven); University Medical Centre Groningen (A.H. Maass); Radboud Universiy Medical Centre (M.A. Brouwer); University Medical Centre Utrecht (M. Meine, A.E. Tuinenburg); Vlietland Hospital (H.A.M. Spierenburg); VU University Medical Centre (C.P. Allaart).

# **List of collected parameters during the registry**

| **Collected Parameters** | **Baseline** | **Every 6 months** | **Every 12 months** |
| --- | --- | --- | --- |
| Informed consent | X |  |  |
| Demographics | X |  |  |
| Aetiology HF | X |  |  |
| Myocardial infarction | X |  | X |
| Cardiologic interventions | X |  | X |
| HF hospitalisation | X |  |  |
| Vascular disease | X |  |  |
| CVA/TIA | X |  |  |
| Cardiac risk factors* | X |  | X |
| Medication† | X |  | X |
| LVEF‡ | X |  | X |
| RFF‡ | X |  | X |
| (Non-invasive) coronary imaging‡ |  |  |  |
| ECG‡ | X |  | X |
| Laboratory‡§ | X |  | X |
| Device data | X |  |  |
| Anti-tachycardia settings | X | X |  |
| ICD deactivation |  |  | X |
| Number of ICD interrogations |  | X |  |
| Documented non-sustained VT’s | X | X |  |
| Sustained VT’s in the monitor zone |  | X |  |
| Arrhythmias treated with ATP |  | X |  |
| Cardiovascular hospitalisation |  |  | X |
| Out-patient visits |  |  | X |
| ICD shock therapy¶ |  | X | X |
| ICD related complication¶ |  | X | X |
| Death¶ |  | X | X |

*HF* heart failure, *CVA* cerebrovascular accident, *TIA* transient ischaemic attack, *LVEF* left ventricular ejection fraction, *RFF* right ventricle function, *ECG* electrocardiogram, *ICD* implantable cardioverter/defibrillator, *VT* ventricular tachycardia, *ATP* anti-tachycardia pacing.

*This includes mitral regurgitation, atrial fibrillation, chronic obstructive pulmonary disease (COPD), diabetes mellitus, hypertension, hypercholesterolaemia, familial sudden cardiac death, smoking.

†This includes the use of beta-blockers, diuretics, angiotensin-converting enzyme (ACE) inhibitors, angiotensin II receptor blockers (ARBs), aldosterone antagonists, platelet aggregation inhibitors, coumarin derivatives, new oral anticoagulants (NOACs), antiarrhythmic drugs, digoxin, non-dihydropyridine or dihydropyridine calcium channel blockers, nitrates, ivabradine.

‡These examinations were conducted as part of routine clinical care. When these examinations were conducted, the data were recorded.

§This includes estimated glomerular rate (eGFR), sodium, potassium, blood urea nitrogen, N-terminal prohormone of brain natriuretic peptide (NT-proBNP), haemoglobin.

¶When the patient experienced an ICD related complication, shock therapy or died at any time during follow-up, an ICD complication form, episode form or a death form was completed.

# **Economic Evaluation Analysis**

The cost-minimisation of targeted supply of implantable cardioverter-defibrillator (ICD) implantation will be performed by health care provider and insurer perspectives with a time horizon of ±2.5 years while discounting the costs beyond the first year. The comparator will be primary prevention ICD implantation in accordance with current practice guidelines [1, 2]. The primary outcome will be the costs of cardiovascular treatment per patient, including all relevant medical costs. Resource use data will be retrieved from the study and unit cost sources will be derived from a common price year, to be determined at time of analysis. The economic evaluation will be done with health economic modelling, using, in particular, decision tree analysis.

Incremental savings will be calculated as the extra savings per screened patient by subtracting the mean costs per patient under the current guidelines from the costs per patient under the application of the new prediction rules. A Kaplan-Meier ‘survival’ analysis approach will be applied with costs instead of time as the continuous variable in order to account for censored costs observations during follow-up. Bootstrapping will be performed to account for sampling variation.

Univariate and multivariate sensitivity analyses will be performed for plausible ranges of unit costs of ICD implantation (plus or minus 10% of the estimated unit costs, see below), for different cost discounting rates (0%, 3.5% and 4%), and for the probability of procedural complications among presumed unnecessary ICD implantations. Subgroup analyses will be made for patients with low, high and very high risk of sudden cardiac death, according to their predicted score from the multivariable clinical prediction model.

Scenario-analyses will be conducted based on alternative cut-off values of the multivariable prediction rules of (i) death without appropriate ICD therapy and (ii) survival for a lengthy period without receiving appropriate ICD shocks. Stated otherwise, we will assess different scenarios in which 0-2% (by increasing steps of 1% at a time) of patients with a predicted hazard for ICD implantation would not receive ICD implantation and see how many extra unnecessary ICD implantations could be foregone.

The budget impact on the mid-term (up to four calendar years) of targeted supply of ICD implantation will be assessed from governmental, insurer and hospital care provider perspectives, in accordance with a recent ISPOR guideline [3, 4]. The budget impact analyses will be prevalence-based, meaning that the yearly estimates include the hospital care expenditures of patients eligible for ICD implantations during the year, whether they present themselves as new patients or as patients with a postponed need for ICD implantation under the developed prediction rules. The analyses will further be patient- rather than episode-based, covering all health care costs observed during follow-up. Considering that the four-years’ time horizon of this budget impact assessment is longer than a patient’s follow-up, additional health economic modelling will be performed based on a conjoint approach of expert knowledge, literature data, and curve fitting techniques to extrapolate costs patterns over time beyond the maximum observation period. The decision tree analysis approach for the cost-minimization analysis will be adjusted and extended for this purpose to include events that may happen during later follow-up.

Epidemiological data on the yearly incidence trends of patients presenting with the target condition in the Netherlands will be derived from national health care surveillance of primary PCI for ST-elevation myocardial infarction and used for projection of future numbers in case of assessment of budgets from a governmental and insurer perspective. Similarly, per hospital yearly production trends for ICD implantations will be used for projections of future numbers in case of budget impact analysis from the hospital care provider perspective. These yearly numbers will be linked to the patient-based cost outcomes to derive yearly budget impact estimates.

Against the base case scenario of following current guidelines by ACC/AHA/ESC [1, 2], we will assess the impact of an instant shift, gradual shift (25% instantly with an additional 25% per subsequent year), or partly instant shift (50%) to targeted supply of ICD implantation. Sensitivity analyses will be performed for the unit costs of ICD implantation (based on the range of observed unit costs), for the type of unit costs (actual costs versus charges), for different levels of allowed false negative rates (0%, 1%, 2%), and for uncertainties concerning epidemiological model parameters.

# **References**

1 McMurray JJ, Adamopoulos S, Anker SD, et al. ESC Guidelines for the diagnosis and treatment of acute and chronic heart failure 2012: The Task Force for the Diagnosis and Treatment of Acute and Chronic Heart Failure 2012 of the European Society of Cardiology. Developed in collaboration with the Heart Failure Association (HFA) of the ESC. Eur Heart J. 2012;33:1787-847.

2 Yancy CW, Jessup M, Bozkurt B, et al. 2013 ACCF/AHA guideline for the management of heart failure: a report of the American College of Cardiology Foundation/American Heart Association Task Force on Practice Guidelines. J Am Coll Cardiol. 2013;62:e147-239.

3 Mauskopf JA, Sullivan SD, Annemans L, et al. Principles of good practice for budget impact analysis: report of the ISPOR Task Force on good research practices--budget impact analysis. Value Health. 2007;10:336-47.

4 Sullivan SD, Mauskopf JA, Augustovski F, et al. Budget impact analysis-principles of good practice: report of the ISPOR 2012 Budget Impact Analysis Good Practice II Task Force. Value Health. 2014;17:5-14.
